# Reproductive competence: a recurrent logic module in eukaryotic development

**DOI:** 10.1098/rspb.2013.0819

**Published:** 2013-09-07

**Authors:** Luke M. Noble, Alex Andrianopoulos

**Affiliations:** Department of Genetics, University of Melbourne, Victoria 3010, Australia

**Keywords:** development, reproduction, competence, vegetative phase change, life history, fungi

## Abstract

Developmental competence is the ability to differentiate in response to an appropriate stimulus, as first elaborated by Waddington in relation to organs and tissues. Competence thresholds operate at all levels of biological systems from the molecular (e.g. the cell cycle) to the ontological (e.g. metamorphosis and reproduction). Reproductive competence, an organismal process, is well studied in mammals (sexual maturity) and plants (vegetative phase change), though far less than later stages of terminal differentiation. The phenomenon has also been documented in multiple species of multicellular fungi, mostly in early, disparate literature, providing a clear example of physiological differentiation in the absence of morphological change. This review brings together data on reproductive competence in Ascomycete fungi, particularly the model filamentous fungus *Aspergillus nidulans*, contrasting mechanisms within Unikonts and plants. We posit reproductive competence is an elementary logic module necessary for coordinated development of multicellular organisms or functional units. This includes unitary multicellular life as well as colonial species both unicellular and multicellular (e.g. social insects such as ants). We discuss adaptive hypotheses for developmental and reproductive competence systems and suggest experimental work to address the evolutionary origins, generality and genetic basis of competence in the fungal kingdom.

## 1. Introduction

Differentiation is the acquisition of specialized capabilities, and development is the overarching ontogenesis. Development unfolds through interplay between the genome, constrained by its evolutionary history, and the environment, to which the genome is adapted. Generation of distinct cell types, a conveniently discrete criterion, is typically considered the minimal unit. Development is induced, meaning simply it is caused by something, and the causes are said to be inducers. Organisms are exposed to the environment over very different scales, however, from the extreme intimacy of a single-celled bacterium to a highly differentiated, homeostatic, long-lived animal. It follows that in more exposed organisms, development is directly induced by certain environmental cues, whereas in more insulated organisms, induction is effected by endogenous cues (hormones) during ontogenesis. Part way along this continuum, many relatively simple organisms use both secreted signals and environmental induction to coordinate development. The response to a fluctuating environment may vary according to homeostatic capacity but is necessarily limited by the metabolic economy (energy), the ultimate determinant of proliferative and reproductive capacity.

In addition to induction and the subsequent response, another fundamental but neglected property of development is competence: the ability to respond to induction [1–3]. In this sense, the term ‘competence’ was introduced into the English scientific literature in 1932 by Waddington, in studying how the vertebrate notochord induces ectoderm to become neural rather than epidermal tissue [4,5]. Waddington outlined three separable aspects of development: competence, the ability to respond to induction; evocation, the inducing stimulus itself and individuation, the resulting process of differentiation.

As for the general terms of differentiation and development, competence can be recognized at varying levels of complexity, from the subcellular (e.g. cell cycle [6] or gene regulatory network [7,8]) to the organismal (e.g. reproductive competence) [9–12]. ‘Competence’ has therefore been applied in different contexts to describe many different processes. Developmental competence could be considered as any measurable state that must precede another in the development of an organism, organ or cell, but a definition encompassing all of ontogenesis at all scales is not useful. More so is the characterization of developmental competence as a ‘checkpoint’ between discrete life-history phases, such as metamorphosis and reproductive maturity.

## 2. Fungal competence

Acquisition of reproductive competence in the model filamentous Ascomycete *Aspergillus nidulans* is a necessary prelude to asexual development, producing dormant spores, and is associated with rapid change in diverse biochemical systems. The existence of competence has also been established in the related pathogen *Aspergillus fumigatus* and in various *Penicillium* and *Trichoderma* species, suggesting conservation in filamentous Ascomycetes at least.

### (a) Reproductive competence in *Aspergillus nidulans*

**Figure 1.**
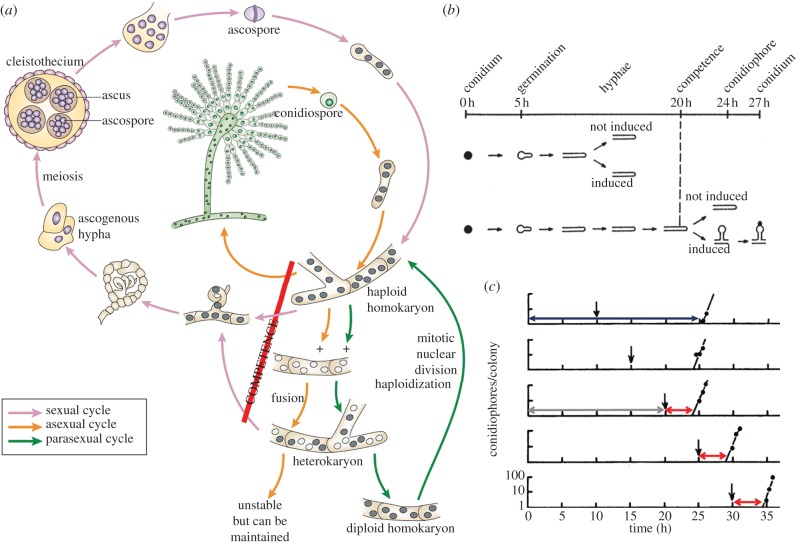
*Aspergillus nidulans* reproductive cycles and the logic of competence. (*a*) Depiction of the four reproductive cycles [13]—asexual, sexual, parasexual (producing recombinant haploid nuclei) and hyphal growth (producing clonal nuclei, although replication errors may contribute to heterokaryosis in a mycelium of sufficient size)—all of which may occur simultaneously in a single colony. (*b*) Developmental timing in *A. nidulans* for a single, isolated conidium growing at 37°C in the presence of excess, preferred nutrients [12]. (*c*) Defining competence in *A. nidulans* [14]. Conidiophores per colony plotted over time since inoculation, in response to variable time of induction (black arrows). Development was induced by exposure of single spore-derived colonies to air and light. Minimum time to conidiation (blue arrow) comprises competence acquisition (grey arrow) and a constant maturation period for conidiophore production (red arrows). Vegetative growth continues indefinitely in the absence of induction and the presence of sufficient nutrients.

*Aspergillus nidulans* reproduction involves some combination of vegetative growth (clonal nuclei distributed throughout the mycelium), asexual development (uninucleate conidiospores), parasexual development (recombinant nuclei via a diploid stage) and sexual development (ascospores via meiosis; figure 1*a*). Conidia break dormancy in the presence of water and an assimilable carbon source with a period of isotropic growth and nuclear division, followed by establishment of a polar growth focus and emergence of a hyphal germ tube [15–18]. Nuclear division is synchronous over the first three to four mitoses (influenced by culture conditions and growth rate), and the first incomplete hyphal barrier, or septum, is laid down around this time [19,20]. Nuclear division within the mycelium subsequently becomes asynchronous, but mitosis and biomass accumulation remain exponential given sufficient nutrients. Actively growing apical septate compartments are highly enriched in nuclei but are also much larger than intercalary compartments, so that the ratio nuclei to cytoplasm remains similar—known as the hyphal growth unit (G). Subsequent subapical branching and mutual hyphal avoidance (negative autotropism) due to accumulation of secreted morphogens leads to the characteristic fungal network, with exploratory growth at the colony exterior and a developmentally committed interior mycelium [21].

Developmental genetic networks have been studied extensively, especially those underlying asexual development, although integration of conserved signal transduction pathways such as ras/cAMP/PKA and G-proteins with fungal-specific transcription factors is rudimentary [22–24]. Diverse inputs ultimately converge on a terminal network comprising transcription factors such as bristle (BrlA) and abacus (AbaA), which establish commitment through feedback loops in the developing conidiophore and drive production of new cell types required for spore generation [25–27].

Without exposure to an inducing air interface and light, however, development is repressed, given nutrient sufficiency. While external nutrient limitation is not a primary inductive cue for differentiation, self-imposed limitation may play a crucial role. Studies of differential gene expression and enzyme activity during conidiation in Ascomycetes demonstrate distinct metabolism in the colony interior and reproductive structures [28–32]. Developmental gene expression is spatially restricted, and ectopic expression of key regulators results in generalized loss of nutrient acquisition and growth arrest [33,34].

Axelrod [12] recognized a minimum of 20 h growth at single colony density is required before *A. nidulans* becomes competent to respond to induction of sporulation (figure 1*b*). Once competent, the period required for production of a conidiophore from vegetative mycelia (known as maturation) is a minimum of approximately 4.5 h at 37°C (figure 1*c*).

In subsequent work, a number of biochemical correlates of competence acquisition were identified. First, the inducibility of two enzymes, nitrate reductase and extracellular protease, falls sharply coincident with developmental competence, while activity of a constitutive enzyme in the pentose phosphate pathway, glucose-6-phosphate dehydrogenase, does not [35]. Thus, it appeared that a change in developmental potential was associated with a pleiotropic change in inducibility of enzymes not themselves required for morphological development. While generalization based on this small sample is fraught with potential for over interpretation, it is possible such a change might occur due to altered transcriptional potential (e.g. chromatin state), protein activity or membrane transport.

Second, intracellular cAMP levels fall sharply during early growth [36]. A role for cAMP-dependent PKA in metabolic activation of spores for diverse fungi is known [16,17,37], and one hypothesis for competence timing is a cAMP cycle determined by conidial deposition. Functions of this conserved chemical messenger in morphogenesis of non-yeast fungi are not well delineated, but gross defects in cell-cycle, sporulation, secondary metabolism, pathogenesis and nutrient sensing have been reported in a range of species [38–40].

Third, media replacement and levels of glucose as carbon and of ammonium or glutamate as nitrogen have no effect on the timing of competence acquisition, although rate of conidiophore production is clearly affected [41]. Competence is therefore stable against external nutrient levels, both high and low, although utilization of non-preferred carbon sources does alter timing (L. M. Noble, A. McLauchlan, K. Hubner, A. Andrianopoulos 2013, unpublished data).

Fourth, and most enigmatic, while acid extraction of competent mycelium results in total hydrolysis of DNA, the same procedure applied to precompetent mycelium results in near complete resistance of DNA to hydrolysis [42]. This result was traced to the interfering action of ferric iron in the mycelial lysate, suggesting ‘an alteration in the concentration, compartmentalization, or species, of iron binding components’ [42]. In all, these gross changes in diverse processes suggest competence acquisition is a time of systemic transition, but do not yet coherently implicate any single underlying cause or mechanism of transduction.

### (b) *Aspergillus nidulans* competence mutants

Axelrod and others employed a simple screen to identify *precocious* competence mutants [12,14]. Subsequent studies showed the effect size in two (genetically uncharacterised) mutants was proportional to glucose uptake rate [43,44]. Specifically, precociousness correlated with both maximum rate of glucose uptake after germination and with the rate of transport decline as competence approached. Fructose, sucrose, alanine and the non-metabolizable glucose analogue 2-deoxyglucose showed a similar uptake rate decline in wild-type, whereas acetate did not. The carbon catabolite derepression system, which hierarchically controls utilization preferences, also differed markedly between states. Precompetent mycelium grown on glucose was derepressed for fructose transport by starvation more rapidly and to a maximal level four to five times higher than that of competent mycelium.

In sum, this work illustrated a relationship, not necessarily a causal one, between nutrient uptake and competence. It is nonetheless tempting to speculate that competence in fungi, as in other organisms, involves a nutrient accumulation threshold. Axelrod's [12] observation that inhibition of protein synthesis produces a dose-dependent delay in competence, yet has little effect (initially) on the subsequent production of conidiophores indeed suggests a greater level of developmental autonomy after competence acquisition.

While no *precocious* genes have been cloned, a second class of competence mutants, temperature sensitive *aconidials*, identified multiple subunits of the COP9 signalosome (CSN), a highly conserved protein complex with homology to the proteasome and eukaryotic initiation factor 3 complexes [45–49]. Mutations affecting ras protein activity also modulate, positively and negatively, competence timing and other developmental transitions [50].

### (c) Ascomycetes: *Trichoderma* and *Penicillium* species

**Figure 2.**
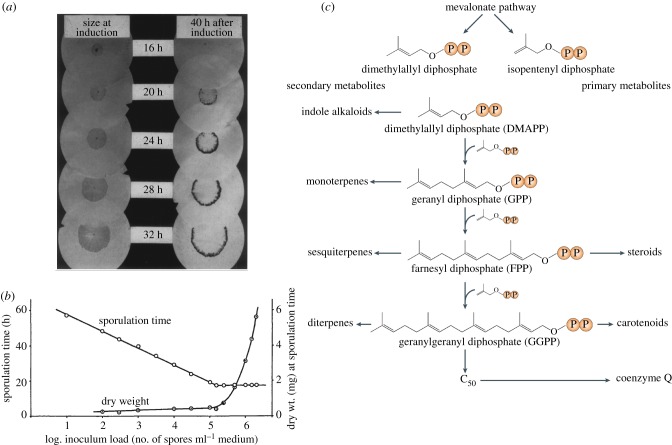
Reproductive competence in *Trichoderma* and *Penicillium* species. (*a*) The relationship between age and conidiation capacity in *Trichoderma viride* [52]. At left, colonies grown on filter papers were fixed and stained with cotton blue at time of induction by light. At right, duplicate photoinduced colonies after a further 40 h incubation in the dark. A ring of conidia is visible at the colony fringe for colonies of age 20 h or greater. (*b*) Identification of density dependence for development of *Penicillium notatum* (*chrysogenum*) [53]. Inoculum load (conidia per ml of medium) is plotted against sporulation time (induced by addition of calcium) and dry weight at time of sporulation. A minimum of 17.5 h is required under these conditions for sporulation. In other experiments, it was found that 6 h is required for conidiophore production (maturation), regardless of inoculum load. (*c*) The mevalonate pathway produces key primary and secondary metabolites in diverse species [54]. The diterpenoid competence hormone, conidiogenone, from *Penicillium cyclopium* is an effector of both competence acquisition and conidiation induction in this species [55].

*Trichoderma* (*Hypocrea*) species have a long history of study, primarily due to antagonistic relationships with plant pathogenic fungi. Developmental competence in this genus refers to two, possibly distinct, phenomena. First is the variable ability of hyphae within a colony to respond to photoinduction of conidiation [51]. The second, unstudied beyond its initial description, is directly analogous to that of *Aspergilli* in that a minimum period of growth is required before ‘accepting’ photoinduction (figure 2*a*) [52].

Research into secondary metabolite production in *Penicillium* species found that sporulation could be induced in liquid culture under certain conditions [56]. The addition of calcium ions was able to fully mimic an inducing air interface, and for *Penicillium notatum*, ‘initial sporulation time varied inversely with … the inoculum load’ (figure 2*b*; [53, p. 419, 57,58]). ‘Mature’ medium, which had supported growth of various fungi, advanced competence acquisition, in some cases to a greater extent than that from *P. notatum* itself.

This body of research lead to isolation from *Penicillium cyclopium* of the only known fungal competence hormone [59]. Related diterpenoid molecules were identified, probably derived from the mevalonate pathway responsible for key hormones across all kingdoms of life, named conidiogenol and conidiogenone (figure 2*c*). Although calcium lowered the effective threshold, conidiogenone alone was able to accelerate competence acquisition and also, surprisingly, induce sporulation. This suggests both competence and induction are hormone thresholds, reporting organism density and emergence into an aerial environment where diffusion is limited [55].

## 3. Competence by any other name?

Notable work on reproductive competence has been carried out in plants (vegetative phase change; [60–62]) and mammals [63–67]. The amoeba *Dictyostelium discoideum* is a special case, with multiple competence thresholds identified at cellular and multicellular levels throughout its experimentally tractable life history. We next discuss competence systems in plants and *Dictyostelium* occupying an equivalent life-history stage to fungal reproductive competence.

### (a) Vegetative phase change in plants

**Figure 3.**
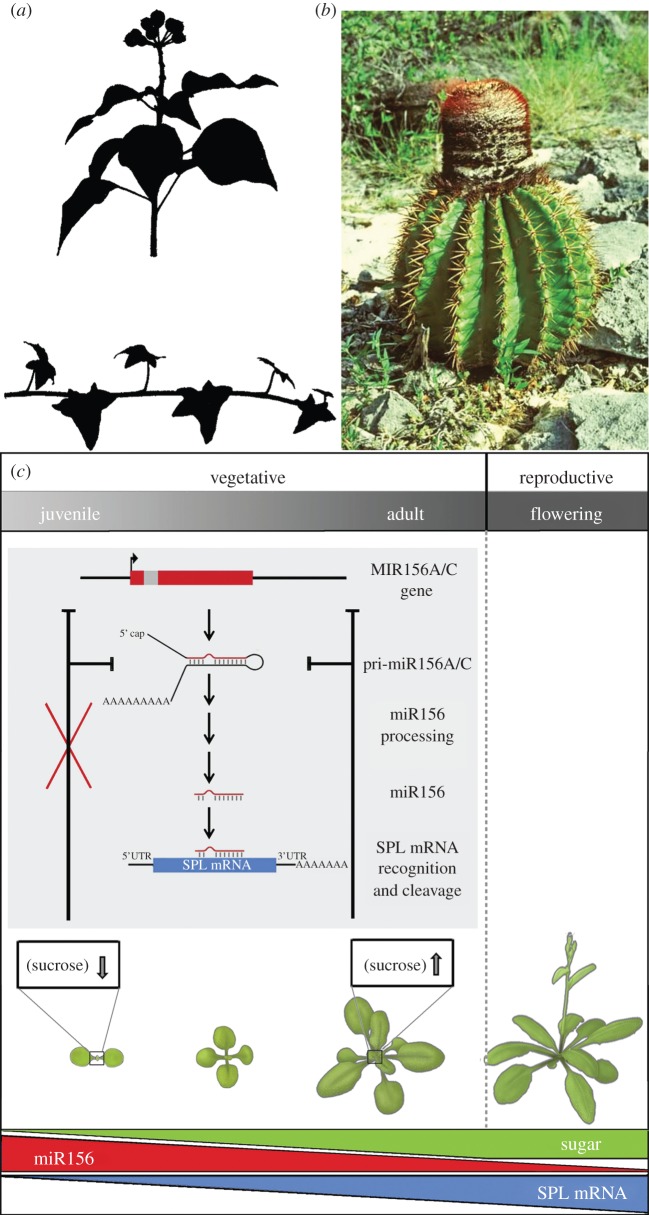
Vegetative phase change and transcriptional regulatory networks. Two examples of distinct morphologies associated with vegetative phases in plants. (*a*) Growth habit and leaf shape in English ivy (*Hedera helix*) for juvenile (lower) and adult (upper) phases [68]. (*b*) *Melocactus intortus* juvenile body (lower) and (upper) the adult apical cephalium [69]. (*c*) Conserved transcriptional networks controlling phase change in plants [70]. Sugars regulate levels of microRNA miR156, which in turn determines expression of the SPL family of transcription factors required for phase change and flowering.

Most developmental research in spermatophyte plants has focused on genetic networks that regulate flower development. Between germination and seed production, however, plants also switch from juvenile to morphologically distinct, reproductively competent, mature vegetative growth [11,60,62]. Phase change is particularly obvious in woody plants, such as English ivy and some cacti, where the juvenile phase is protracted and distinctive in growth form (figure 3*a*,*b*) but was perhaps first documented in the course of fruit-tree grafting [68]. It has now been studied at the molecular level in *Arabidopsis*, maize and other species, addressing the measurement of time during development and the environmental and endogenous signals involved in regulating transitions.

Basic factors such as photoperiod and temperature, critical as they are to carbon metabolism and environmental forecasting, play an important role in plant competence acquisition [71]. The diterpenoid gibberellin is another important factor. Although genetic networks peripheral to this hormone in phase change are not well understood, biosynthetic and hypersensitive mutants show altered onset of maturity [72,73]. Most illuminating, numerous studies have implicated a conserved role for RNA silencing [74–77]. MicroRNA miR156 has been identified as a crucial control point through targeting the *SPL* (SQUAMOSA promoter-binding protein-like) family of transcription factor genes [78]. These factors, in turn, enable flowering and establish mature epidermal identity by activating further activators and repressors of repressors, including miR172 [61,77]. The inverse expression pattern of these two miRNAs, with miR156 abundant in juvenile shoots but decreasing over time, and negative feedback from their cognate transcription factor targets, underlies the bistable morphologies and reproductive capabilities associated with the phases (figure 3*c*). Recent work suggests that sugars regulate miR156 levels [79], and that gibberellin and miR156 signalling may be integrated at the level of SPL factor regulation [80], providing a direct link between central carbon metabolism and phase change.

### (b) Dissecting *Dictyostelium* development

Historically grouped with fungi, soil-dwelling slime moulds are now placed within a sister clade to the Opisthokonts, the Amoebozoa [81], and most recent phylogenomic analyses merge these into a monophyletic supergroup, the Unikonts [82]. As for plants and fungi, the earliest stages of ontogenesis for *D. discoideum* most relevant to reproductive competence acquisition have received little attention relative to the later spectacular transition to multicellularity for which the model organism is most famous. *Dictyostelium discoideum* is in fact highly derived among Amoebozoa [83], many of which develop only by encystment or production of very simple fruiting bodies—several clades produce a spore and crude stalk from a single cell. As for prokaryotes with similar reproductive modes, such as myxobacteria [84–86], development in *Dictyostelium* is a direct response to starvation and high population density, mediated by quorum signals and other secreted morphogens [87–90]. cAMP released from amino acid-starved cells above a critical density settles into synchronized oscillations throughout the population, inducing polarization and chemotaxis towards a central organizing centre. After sufficient exposure, cells become ‘aggregation competent’ [91], forming a migratory slug that searches for favourable fruiting conditions and, eventually, an upright structure promoting spore dispersal.

Competence to respond to starvation is dependent on constitutively produced autocrine signals [92]. At least three are known, termed prestarvation factors (PSFs), though the responsible genes are not. At a threshold PSF: bacteria (food) ratio, production of another morphogen, conditioned medium factor, leads to transcriptional induction of a small number of pivotal genes, including those for the cAMP receptor and a heterotrimeric G-protein through which cAMP signalling is propagated [93].

Interestingly, this competence threshold is not always absolute—spores of at least one species are able to directly re-enter development without a period of feeding, owing to an unusual positive relationship between spore density and germination (the relationship is negative in other *Dictyostelids* and in many fungi). This could be reiterated, producing successive generations of progressively smaller cell size, indicating spore metabolic reserves are not strictly limiting [94].

## 4. Questions of mechanism and generality

A threshold governing reproductive capacity is a common feature of development, whether of prokaryotes or eukaryotes, single-celled or multicellular. In *D. discoideum*, competence checks govern multiple transitions from a population of single cells to a cohesive multicellular entity. Quorum sensing molecules (typically divergent, with the notable exception of cAMP) are channelled into conserved signal transduction pathways (e.g. G-proteins) to coordinate behaviour. This includes the earliest stage of vegetative growth, comparable with competence in fungi, where signals reporting organism density and nutrient availability are integrated to determine the switch from niche exploitation to reproduction and dispersal. Competence thresholds in plants and other Unikonts are mediated by intercellular signals and nutrient acquisition. The apparent broad conservation of mechanisms invites several questions and suggests lines of enquiry to compare basal genetic underpinnings, within fungi and more broadly.

### (a) How does fungal reproductive competence relate to known mechanisms of spatio-temporal differentiation?

Metabolism in fungi is often discussed as a singular phenomenon, research being grounded on homogeneous liquid culture and unicellular yeasts. Classical studies of the filamentous growth form, developmental studies (which usually must embrace heterogeneous solid culture) and research aimed directly at measuring spatial differentiation within the mycelium illustrate a different picture [15,19,29,95,96]. This may be of relevance for competence acquisition, with intra-hyphal specialization of anabolism within the mycelium interior, and catabolism, secretion and sensory integration at the hyphal apex [21]. The relationship between this hypothesis and established mechanisms of polarity establishment and maturation during early growth remains to be clarified [20,97–100]. Among early changes in hyphal differentiation are the demarcation of hyphal compartments by septa and establishment of apical-high gradients of reactive oxygen species, Ca^2+^ and protons [96,97,101,102]. It would be of interest to monitor these landmarks temporally to see if their establishment coincides with competence acquisition or is altered in mutants.

For sessile organisms on solid substrate, autoinducers accumulate as a predictable function of density and distribution and report physical aspects of the environment such as diffusion [103]. Competence in fungi is density dependent, even when nutrients are maintained in excess [41]. This presumably means that multiple spores can more rapidly form a mycelium equivalent to that of an older colony derived from a single spore, and so density approximates age. A large and growing number of autoregulatory substances secreted by mycelial fungi are known, including oxylipins—lipidic signalling molecules found across all eukaryotic kingdoms [104–107]—fungal-specific secondary metabolites [108] and growth by-products such as bicarbonate ([92], reviewed in [105]). With the exception of the diterpenoid conidiogenone produced by *P. cyclopium* [59], none are known to alter competence timing. Competence in fungi may be sensitive to both specific morphogens and general effects of environment modification as an inevitable consequence of growth, such as pH.

### (b) How is nutrient acquisition related to competence acquisition?

Evidence for reproductive competence acquisition as a nutrient accumulation threshold across diverse organisms is strong. In humans, fat and leptin levels predict sexual maturity better than chronological age [63,65,109]. Many plants require a critical biomass accumulation before flowering can be induced [110]. Age at maturity is negatively correlated with growth rate in insects [111], and similarly, moulting and metamorphic competence is governed by a critical weight threshold [112–114].

Competence in *A. nidulans* is indeed dependent on metabolism based on (i) correlation with glucose uptake rate, (ii) temperature dependence and (iii) slower acquisition on non-preferred carbon sources (although excess nutrients do not accelerate competence, suggesting metabolic saturation). However, the threshold does not appear to represent some absolute minimal attainment of nutrients for successful reproduction. *Penicillium cyclopium* exposed to the competence hormone conidiogenone from inoculation is able to undergo ‘microcycle’ conidiation shortly after germination [59], and the dictyostelid *Dictyostelium stoloniferum* is able to cycle though multiple generations without intervening growth [94]. Reproductive competence thresholds even in relatively simple eukaryotes may therefore represent constrained but variably plastic, optimized trade-offs rather than absolute limits.

### (c) Is competence acquisition always adaptive?

Maintenance of discrete growth phases in plants is considered adaptive [60,68,115], citing, for example, expression of distinct resistance mechanisms where predators and parasites vary between phases as a plant colonizes the aerial niche [116]. Adaptive hypotheses have also been proposed for metamorphic competence in marine invertebrates [117–119] and holometabolous insects [120], and sexual maturity in mammals [9]. This may be true, but may also be no more than *post hoc* justification according to ecology, without bearing on why a competence state first arose.

Life forms adapt to their environment, within the genetic, morphological and behavioural constraints set by population dynamics and evolutionary history. We suggest reproductive competence is an elementary logic module, which by necessity has been elaborated upon to allow coordinated development of multicellular organisms or functional units. This includes unitary multicellular life as well as colonial species both unicellular and multicellular (e.g. social insects such as ants, which reproduce colonies only above a critical size [121]). Such behaviour would be expected to emerge from nonlinear dynamics of genetic networks reflecting strong preference for stable, committed development.

The simplest hypothetical ancestral state exhibiting reproductive competence is a single cell capable of producing a single, dormant spore in response to an unfavourable environment, as seen for some bacteria, amoebozoa and yeasts. Here, near the upper extreme of environmental dependence, reproductive competence must be tightly integrated, if not synonymous, with the cell cycle: a phase of nutrient acquisition and biomass accumulation, followed by DNA replication and differentiation of a distinct cell type. Tracing this ontogenic phase back in time with evolutionary developmental studies would reveal whether competence in multicellular organisms shares genetic heritage with the cell cycle. In this regard, the finding that ras activity controls developmental transitions, nuclear division and polarity in *Aspergilli* [50,99,123], and the essentiality of the CSN in regulation of both competence and the cell cycle warrants further attention [123,124].

Beyond evolutionarily related reproductive competence states where conservation of underlying genetic architecture could be expected, other thresholds may share only conceptual homology due to emergent organization [125]. In other words, competence thresholds are simply necessary for efficient multicellular development, as seen most clearly in the multiple competence states delineated during development of *D. discoideum* from independent, single cells through a multicellular, motile aggregated stage to construction of a fruiting body for dispersal of dormant spores. Achievement of each state requires coordinated signalling, and competence in at least one case is as simple as expression, or not, of a single gene, the receptor for cAMP. So competence may be adaptive, in the sense that coordinated development is obviously adaptive, but competence systems in different organisms may be adapted to their current respective niches to very different degrees.

In the case of fungi, there is little research on competence generally, and certainly no evidence that timing is an ecologically relevant, selected trait. This is in contrast to, for example, the widespread phenomenon of conidial autoinhibition (density-dependent inhibition of germination), which can at least be suggested as a plausible mechanism to promote dispersal and reduce competition [126], or circadian rhythmicity for conidiation [127].

If transition points between discrete developmental phases are optimized thresholds, whether of nutrient acquisition or environmental sensing (or hormones reporting these cues), questions of what these thresholds physically represent, what deviation from the optimum means for the organism, the population genetic or epigenetic variation for these traits and the relationship to organism ecology remain to be addressed. Answers to these questions in experimentally tractable fungi would require broad population genetic research looking for variation in competence timing among wild-isolates, mechanistic study of the effects of manipulating competence timing on reproductive output, and biochemical and genetic study to determine the factors of relevance for competence timing and the networks underlying their production.

## 5. Summary

Interplay between nutrient acquisition and parental nutrition, coordinated by chemical messengers, is a recurrent theme in development of plants and organisms spanning *Dictyostelids* to mammals. Discrete life phases in Unikonts may trace their roots to early spore-bearing species subject to a strict competence threshold integrated with the cell cycle. The existence of reproductive competence in fungi is well established, though not well appreciated, and its genetic and epigenetic basis, taxonomic distribution and current ecological relevance, if any, are poorly understood. Recent transcriptional profiling of *A. fumigatus* has revealed significant transcriptional rewiring associated with competence acquisition [128], and early biochemical work in *A. nidulans* shows pleiotropic change in membrane transport, intracellular iron availability and other systems. This clearly necessitates controlling for competence state in experimental studies. With amoebozoa such as the *Dictyostelids*, study of competence in a range of fungal species is required to establish its generality and relevance to other more elaborate eukaryotic systems.
